# Interaction Between Virus-Like Particles (VLPs) and Pattern Recognition Receptors (PRRs) From Dendritic Cells (DCs): Toward Better Engineering of VLPs

**DOI:** 10.3389/fimmu.2020.01100

**Published:** 2020-06-09

**Authors:** Jesús Zepeda-Cervantes, Josué Orlando Ramírez-Jarquín, Luis Vaca

**Affiliations:** ^1^Departamento de Biología Celular y del Desarrollo, Instituto de Fisiología Celular, Universidad Nacional Autónoma de México, Mexico City, Mexico; ^2^Departamento de Neuropatología Molecular, Instituto de Fisiología Celular, Universidad Nacional Autónoma de México, Mexico City, Mexico; ^3^Department of Physiology and Biophysics, University of Washington School of Medicine, Seattle, WA, United States

**Keywords:** viruses, epitopes, tropical viral diseases, allergy, cancer, translational medicine, personalized medicine

## Abstract

Virus-like particles (VLPs) have been shown to be strong activators of dendritic cells (DCs). DCs are the most potent antigen presenting cells (APCs) and their activation prompts the priming of immunity mediators based on B and T cells. The first step for the activation of DCs is the binding of VLPs to pattern recognition receptors (PRRs) on the surface of DCs, followed by VLP internalization. Like wild-type viruses, VLPs use specific PRRs from the DC; however, these recognition interactions between VLPs and PRRs from DCs have not been thoroughly reviewed. In this review, we focused on the interaction between proteins that form VLPs and PRRs from DCs. Several proteins that form VLP contain glycosylations that allow the direct interaction with PRRs sensing carbohydrates, prompting DC maturation and leading to the development of strong adaptive immune responses. We also discussed how the knowledge of the molecular interaction between VLPs and PRRs from DCs can lead to the smart design of VLPs, whether based on the fusion of foreign epitopes or their chemical conjugation, as well as other modifications that have been shown to induce a stronger adaptive immune response and protection against infectious pathogens of importance in human and veterinary medicine. Finally, we address the use of VLPs as tools against cancer and allergic diseases.

## Introduction

Virus-like particles (VLPs) have attracted the attention of many researchers, most significantly virologists and immunologists. VLPs are supramolecular structures that closely resemble viruses (analogous to an empty shell), but are replication-deficient because they contain no viral genetic material (DNA or RNA) (1, 2). VLPs can directly interact with antigen presenting cells (APCs). Dendritic cells (DCs) are the most potent APCs (3–6).

Overall, VLPs are sensed by pathogen recognition receptors (PRRs) which are expressed on the surface (1, 7) or endosomes (8–10) from DCs. VLP uptake prompts DC maturation and presentation of peptides loaded into major histocompatibility complex (MHC) class I or class II molecules for priming CD8^+^ or CD4^+^ T cell responses, respectively. CD4^+^ T cells help B cells to produce antibodies (Th2 cells) and enhance CD8^+^ T cells (Th1 cells) cytotoxicity (11–15). Since VLPs contain multimeric epitopes on their surface, they can promote cross-linking of B cell receptors (BCRs). Simple cross-linking of BCRs by VLPs can be strong enough for priming B cells and induce the production of antibodies even without the help of CD4^+^ T cells. B cells can work as APCs, taking up VLPs, processing and presenting them to T cells (2, 4, 12, 16–24). Therefore, VLP-based vaccines are capable of inducing both humoral and cellular immune responses, and due to their multimeric nature, adjuvant co-administration is not needed in most cases, but the use of adjuvant improves their immunogenicity.

The aim of the present review is to summarize the findings regarding the interactions between VLPs and pattern recognition receptors (PRRs) from DCs, in an attempt to highlight avenues directed to the more efficient VLP design.

## VLP Definition

VLPs are supramolecular assemblies with the same or similar structure as native virions of about 10–200 nm in diameter (12, 22, 24–26). VLPs are made up of copies of one or more viral proteins that self-assemble into nanoparticles. In addition, VLPs do not contain genetic material and thus are not infectious for the vaccinated individuals. For this reason, VLPs are safer than whole-pathogen based vaccines such as those containing attenuated viruses (2, 19, 22, 23, 25–32). Due to the above, VLPs do not have the drawback of replicating, recombining, reassortment or reverting to virulent stage as may occur with traditional vaccines (33, 34). In terms of immunity, VLPs represent pathogen associated molecular patterns (PAMPs) due to their multimeric structures with conformation that resemble that from wild type viruses. VLPs can be discriminated against by the host, and therefore may induce an adaptive immune response (35).

## Synthesis and Design of VLPs

VLPs can be produced by heterologous expression systems such as yeast or baculovirus (1, 36–38) although other systems such as plants or bacteria have been also used (8, 39–41). However, the last one does not support protein glycosylation (24), a post-translational modification that may be required to generate an adequate immune response (24, 42–45). Mammalian cells can also be used as expression system, which allows complex post-translational modifications but with the drawback of having very low yields (0.018–10 μg per ml) when compared to bacteria or baculovirus expression systems (24).

VLPs can be used as an antigen delivery system by themselves but they can also carry individual epitopes from other pathogens (46–48). First, for the conventional VLP synthesis, capsid proteins must be expressed in an efficient protein expression system to reach high yields of the desire VLP (2, 25, 29). Second, fusion proteins can be designed by using capsid proteins to synthesize chimeric VLPs by incorporating immunorelevant exogenous epitopes (2, 19, 25, 29, 49, 50). This is a method very useful for displaying exogenous antigens on VLP surfaces (51, 52). However, the fusion of exogenous epitopes to capsid proteins could destabilize the VLP structure or interfere with protein and/or epitope folding. A well-designed chimeric VLP can be generated by inserting the target epitope into loop structures of the VLP protein (2, 53). It is preferable that exogenous epitopes range from 20 to 30 amino acids, avoiding the insertion of positive amino acids or with high hydrophobicity (54) as well as avoiding amino acids with tendency to form β-strands because it could interfere with the self-assembly process of the VLP (19, 54). And third, chemical attachment of small peptides or even full-length proteins can be used to incorporate exogenous antigens of specific pathogens on VLPs (18, 40, 55). In this case, VLPs and target antigens are produced separately and conjugated at a later stage. These are the most frequent forms used to incorporate exogenous antigens to the surface of VLPs. Conjugation can be covalent or non-covalent. The chemical attachment has the advantage that full-length proteins can be bound to VLPs. VLPs used commonly with this strategy are based on MS2 and Q-beta bacteriophages (8, 9, 18, 39, 56–58). A strategy of non-covalent conjugation includes the biotinylation of VLPs and exogenous antigens and the use of streptavidin which acts as a linking molecule (19, 59, 60). The mutation of an amino acid to produce an exposed cysteine on VLP surface and the addition of an antigen containing a maleimide group that can react with sulfhydryl groups is an example of covalent conjugation (61).

It is also important to know that some VLPs can elicit a stronger immune response than others and this fact is a very important point when choosing the VLP. The hepatitis B virus (HBV) core antigen (HBcAg) dimerizes and forms spikes found in the surface of HBV, and it has been used as a strategy for displaying exogenous antigens to increase their immunogenicity (62). For instance, six amino acids from the protein gp41 from human immunodeficiency virus (HIV-1) were displayed on HBV VLPs and mice immunized with these VLPs produced high antibody titers against gp41, although these antibodies were not neutralizing (34, 63). The same epitope was more successful when it was displayed on VLPs formed by influenza A proteins and in this case neutralizing antibodies were produced (34, 63). This is a clear example of how important the selection of the VLP is. Typically, one must match the VLP selected with the disease to be treated. In many cases this selection involves testing several combinations to find the one that produces the best immune response.

## Commercial VLP-Based Vaccines Against Human and Animal Viruses

The first VLP-based vaccines were designed because of some viruses cannot replicate efficiently in cell culture and VLPs showed to be better than traditional vaccines in terms of safety (64). Currently, some VLPs have been approved for human vaccines. The first commercialized VLP-based vaccine was against HBV. At the beginning of the vaccine formulation, empty particles of HBV containing the surface antigen (HBsAg) were obtained from blood of infected individuals (34, 65–67). Although these empty particles could be purified, the yield of antigen was limited and this hindered their wider use. Later, the cDNA encoding for HBsAg was inserted in yeast cells by means of the recombinant DNA technology and it was shown that HBsAg self-assemble into VLPs (34, 67, 68).

The commercial vaccines Engerix® (GlaxoSmithKline) and Recombivax HB® (Merck & Co) against HBV were approved in the 1980s and they are based on VLPs (19, 69). The second commercially available VLP-based vaccine was Gardasil®, approved in 2006 to prevent human papillomavirus (HPV) infections (19, 70). This vaccine contained 2 different genotypes (HPV-16 and HPV-18), which correlated strongly with the appearance of cervical cancer (34, 62).

The HPV capsid is formed by two proteins, L1 and L2. L1 is the major capsid protein and its single expression or with L2 lead to the generation of 40-nm VLPs (19, 70). Although L1 contains neutralizing epitopes, these epitopes are not conserved among other HPV types. Contrarily, L2 cannot form VLPs on its own, but it is highly conserved and anti-L2 antibodies provide protection against several HPV types (62). It has been strongly suggested that this vaccine reduces or prevents the development of intraepithelial neoplasia as indicated by neutralization assays (2, 34, 62, 71).

Cervarix® (GlaxoSmithKline) and Gardasil® (Merck & Co) are commercial vaccines against HPV. Cervarix® protects against HPV types 16 and 18 whereas Gardasil® protects against HPV types 6, 11, 16, and 18 (29, 71–73). Gardasil-9® protects against the pathogenic HPV types: 6, 11, 18, 31, 33, 45, 52, and 58 (62). HPV VLPs induce immune response without adjuvant co-administration, but the adjuvant use can improve the protection against genetically related virus types (34, 74). All commercially available HPV vaccines based on VLPs are co-administrated with adjuvant, Gardasil® contains aluminum (72) whereas Cervarix® contains adjuvant system 04 (AS04) (71, 73).

VLPs are also being used in veterinary medicine. The first commercially available veterinary vaccine based on VLPs was against porcine circovirus type 2 (PCV2) (2, 75). PCV2 generates economic problems around the world in the pig industry (76) causing several syndromes in all stages of pig growth (77). The traditional vaccines against PCV2 are based on inactivated PCV2 or a chimeric PCV1/2 (a PCV that contained the backbone DNA of PCV1, a non-pathogenic PCV, but the ORF2 protein from PCV2). Even though it was previously reported that ORF2 protein from PCV2 self-assembled into capsid-like particles (78), Circoflex® (Boehringer Ingelheim), Circumvent® (Intervet/Merck) and Porcilis PCV® (Schering-Plough/Merck) were introduced to the market 6 years later as the first veterinary vaccines based on VLPs (75, 76). The PCV2 vaccines based on VLPs have been shown to generate neutralizing antibodies which are directed against ORF2 epitopes (76).

There are other prototype vaccines based on VLP tested in veterinary medicine. VLPs of porcine parvovirus (PPV) have been used as antigen delivery system and they are based on the expression of VP2 capsid protein which self-assembles into 25-nm VLPs (2, 46–48). Immunization with these VLPs induces both humoral (46, 47) and cellular immune responses (46), does not need adjuvant co-administration and can be used alone or with exogenous epitopes fused to VP2 protein (46).

A pathogenic virus that infects mammalian species including both humans and animals is rotavirus which causes diarrhea (79). Rotavirus VLPs can be generated by expressing viral proteins 2, 4, 6, and 7 and they can confer cross-protection in several animal species (2, 80, 81). Another example of pathogenic virus is the rabbit hemorrhagic disease virus (RHDV) which cause hepatitis and hemorrhages (82). The capsid from RHDV is a 40-nm nanoparticle formed by 90 dimers of VP60 that self-assemble (82, 83). RHDV VLPs can be used for designing chimeric VLPs inserting a foreign epitope at the N-terminus of the RHDV capsid protein. These VLPs induce the expression of interferon-γ (IFN- γ) and a cytotoxic T-lymphocyte (CTL) response (2, 83).

**Figure 1:**
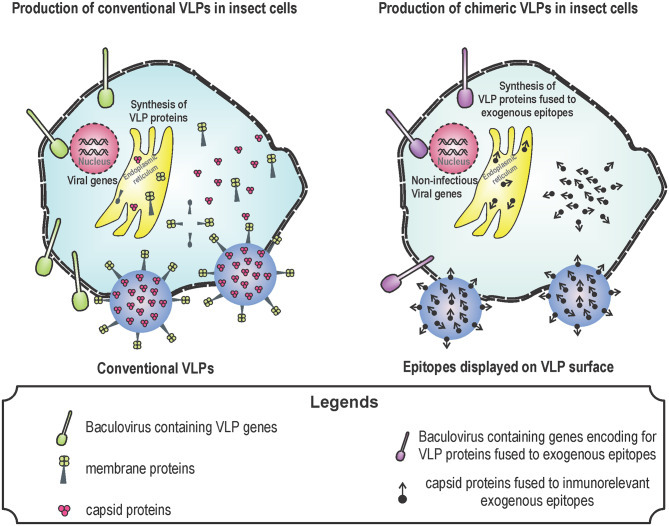
Production of virus-like particles (VLPs) in baculovirus system. Baculovirus system is one of the most used for the production of VLPs. The design of VLPs is based on the expression of viral proteins that self-assemble by using genetically modified baculoviruses. These proteins are produced during the infection of insect cells with the recombinant baculoviruses. Baculovirus infection induce the expression of VLP proteins, VLPs are formed by means of self-assembly and VLPs together with baculoviruses leave the cell through lysis or budding. Conventional (left) and chimeric (right) VLPs can be produced for several uses: vaccine against pathogens in the case of conventional VLPs; and vaccines against cancer or allergy in the case of chimeric vaccines.

To generate enveloped VLPs, the structural proteins of the virus must be produced in cells to simulate a natural infection and promote self-assembly of the proteins. As an example, to generate influenza A VLPs, hemagglutinin (HA), neuraminidase (NA), and matrix proteins (M) from influenza are expressed in the baculovirus expression system. Influenza viruses are classified according to HA and NA proteins located in their surface (36, 37). Influenza viruses are antigenically different and there are 18 HA and 11 NA (36). In humans, the most important influenza A subtypes are H1N1 and H3N2 due to their morbidity and mortality (11). All influenza A vaccines are based on HA, because this protein is responsible of the attachment of virus to target cells (11, 14). However, the efficacy of these vaccines against different influenza A strains is low because HA is highly variable (36, 37). VLPs containing tandem epitopes from the extracellular domain of M2 protein from human, avian and porcine influenza A viruses showed better protection than VLPs containing HA alone (36). Newcastle disease virus (NDV) is one of the most important avian pathogens causing respiratory and nervous symptoms. NDV contains the viral proteins: M protein, nucleocapsid protein (NP), F protein, and hemagglutinin-neuraminidase (HN) protein. All these proteins, when overexpressed, self-assemble into VLPs (2, 84). NDV VLPs induce neutralizing antibody titers similar to those raised by immunization with an inactivated NDV. Surprisingly, NDV VLPs induce T-cell responses higher than traditional vaccines (2, 84) (Figure 1 shows the process for synthesizing conventional and chimeric VLPs in insect cells).

The use of algorithms is another strategy that can assist the design of improved VLPs. For instance, COBRA (computationally optimized broadly reactive antigen) was used for the development of VLP-based vaccines against subtypes H1, H3, and H5 of influenza A virus. These vaccines induced antibodies that block HA antigen, and inhibit the infection and pathogenesis of wild type influenza virus in mice (85, 86).

## DC Development

DCs are a heterogeneous population of cells (87). Different types of immature DCs subsets reside in mucosal tissues and skin, such as the epidermal Langerhans cells (LCs) and the dermal DCs which express Langerin (CD207) and DC-SIGN (DC–specific intercellular adhesion molecule 3–grabbing non-integrin, also called CD209), respectively. DCs sense antigens through receptor-mediated endocytosis (4, 42) as occur with Langerin which is a C-type lectin receptors (CLR) involved in the internalization of mannose-containing ligands (88). For *in vitro* generation of DCs, monocytes are cultured with cytokines: granulocyte/macrophage colony-stimulating factor (GM-CSF) and interleukin 4 (IL-4) (89–91) or GM-CSF, IL-4 and transforming growth factor beta 1 (TGF-β1) to generate DCs phenotypically similar to dermal DCs or epidermal LC, respectively (91). LCs and macrophages share a common precursor (92) but LCs also express the transcription factor Zbtb46, that is selectively expressed by DCs (92, 93). In other words, LCs are a type of cell that shares genes of macrophages and DCs but have functions of DCs (92).

The macrophage—dendritic cell progenitor (MDP) is a common myeloid progenitor that differentiates in: monocytoid lineage and common DC progenitors (CDPs), depending on its environment (growth factors, cytokines, and transcription factors). CDPs can be differentiated in plasmacytoid DCs (pDCs) and pre-dendritic cells (pre-mDCs). Pre-mDCs are precursors of CD11b^+^ and CD8α^+^ DCs. mDCs express chemokine receptor 7 (CCr7), zinc finger and BTB domain containing 46 (Zbtb46) and FMS-like tyrosine kinase 3 ligand (Fl3) throughout their development (87). mDCs secrete high amounts of IL-12 (94) whereas pDCs secrete high amounts of IFN type I (called the IFN-producing cells) through activation of transcriptional factors such as interferon-regulatory factor 3 (IRF3) and IRF7 to arrest viral infections, although other types of DC also produce IFN type I (such as IFN-α) but in low quantities (12, 23, 95). DCs derived from monocytes are the most potent APCs (4).

pDCs and CD8^+^ Lymphoid DCs are the most abundant DCs in the lymphoid organs (96, 97). Lymphoid DCs are found in T cell areas in the lymph nodes and thymic medulla, are related to tolerance (90, 98) but recently have been showed that CD8^+^ DCs induce the generation of cytotoxic T cells (23, 48, 98). However, monocytes also give rise to CD8^−^ and CD8^+^ DCs located in the spleen (99). There are also CD4^+^ DCs, but these DCs loss CD4 molecules and express CD8 when activated with VLPs (48).

There are other cells with the DC morphology called follicular DCs which are not properly DCs. Follicular DCs can present antigens on their surface (12, 100) capturing particulate antigens (VLPs and antibody-antigen complexes) which is essential for B cell activation in the lymph nodes (101).

## Early Interactions Between DCs and VLPs

VLPs activate DCs through their interaction with PRRs. PRRs have evolved to recognize PAMPs which are not found in host cells but are present in bacterial, viral, parasitic and fungal pathogens (102–105). VLPs can attach and penetrate host cells because they conserve their receptor binding sites (2, 30, 34). DCs use their PRRs to recognize VLPs in the same way they detect wild type viruses (7, 15, 44, 50, 106–108). After immunization with VLP-based vaccines, VLP and adjuvant are recognized and internalized by DCs, inducing the release of pro-inflammatory cytokines to recruit more APCs which later prompt an adaptive immune response (2, 109).

Some important features involved in antigen uptake by DCs are hydrophobicity and charge. For instance, negatively charged particles are best associated with APCs (23, 110). Similarly, hydrophobic particles are phagocytosed more efficiently (23, 110). The size of antigens also plays an important role in the uptake, activation and presentation of VLPs. Particles from 500 nm to 2 μm are taken up by DCs found in the injection site, which migrate later to lymph nodes, whereas particles ranging from 10 to 200 nm pass directly through lymphatic vessels reaching the lymph nodes where they are trapped by follicular DCs (12, 23, 111).

## Binding of VLPs to DC Receptors

Immature DCs cannot adequately stimulate T cells (5), but can migrate to inflamed tissues (5, 112) to uptake antigens and process them (2, 5, 6, 15, 23, 25, 42, 95). DCs internalize particulate antigens such as VLPs through direct interaction with their PRRs (2, 23, 25). PRRs include CLRs (7, 42, 43, 113–115), Toll-like receptors (TLRs) (1, 2, 9, 10, 15, 24, 34, 57, 58, 60, 87, 95, 99, 102, 109, 111, 116–119) and others like NOD-like receptors (NLRs) (102, 120–122), but PRRs that interact directly with viral proteins are CLRs, TLRs (1, 7, 123, 124) as well as Fcγ receptors (FcγR) found on the surface of the plasma membrane from DCs (15).

CLRs contain carbohydrate recognition domains (CRD) that interact with proteins containing sugars such as mannose or galactose in a calcium dependent manner (42, 43). The main CLRs include DC-SIGN, Langerin, the mannose receptor (CD206), and DEC-205 (CD205) (42, 43). CRDs of CLRs work as antigen receptors that facilitate the binding and uptake of glycosylated ligands. CLRs on DCs are endocytic receptors and some of them are constitutively internalized whereas others are internalized upon ligand binding (42).

DC-SIGN is a CLR expressed on the surface of DC, interacts with mannose residues of glycoproteins through its CRD located in its C-terminal and also acts as an adhesion molecule. DC-SIGN participates in migration of the DC and in the activation of T cells (42). DC-SIGN binds and internalizes several viruses, including HIV-1 through its envelope protein gp120 (42–45, 125). Similarly, HPV VLPs can also bind DC-SIGN, as transfection and interference experiments have demonstrated (44). Another pathogen that can bind DC-SIGN is cytomegalovirus (CMV). CMV can be captured by the DC through its interaction with DC-SIGN, but this facilitates the transmission instead of promoting protection. The glycoprotein B of the membrane from CMV is the ligand for DC-SIGN (106).

Some viruses can bind to different types of DCs and/or use different PRRs as binding sites. HPV VLPs can bind to human DCs and LCs (7, 50) but use different pathways of cell binding (7). In host cells, the first step for binding of VLPs is the interaction with heparan sulfate so that the addition of heparin inhibits this process (107, 126). Heparan sulfate is necessary for the binding of HPV VLPs to DC but not to LC, instead of this, HPV VLPs interact with Langerin in LC as immunofluorescence and colocalization experiments have been shown (107).

Another PRR of immature LCs is the FcγR. FcγR is also expressed on the surface of monocytes, DCs, macrophages, natural killer cells, among others (105, 127, 128) and its main function is to bind the constant region of the heavy chain of immunoglobulins (129). Monomeric immunoglobulin G (IgG) bind to FcγRI to activate an internalization-recycling pathway (130), whereas the cross-linking of FcγRs on monocytes activate the phagocytosis and the release of cytokines (127). Thus, FcγRs enable the interaction of molecules of the adaptive immune system such as IgG with innate immune cells such as DCs and macrophages, leading to the specific recognition of pathogens (131). An important point is that antibodies lacking glycosylation bind poorly to FcγR (128, 132, 133), which indicates that glycosylation play an important role in this recognition. Apart from recognizing antibodies, FcγR can recognize directly some PAMPs: HPV VLPs bind FcγR on DC and this recognition promotes high levels of MHC as well as the expression of costimulatory molecules (15).

## VLP Uptake by DCs

Immature DCs have high endocytic capacity to uptake antigens (4) capturing small particles about 100–500 nm, unlike the macrophages which capture up to 15 μm particles (103). DCs preferably uptake particles around 40 nm whose size corresponds to that of the VLPs (2, 25). In addition to the fact that VLPs are PAMPs, they are also more efficiently taken up by DCs than soluble antigens, thanks to their particulate nature (12, 134). Antigen properties such as: hydrophobicity, hydrophilicity, shape, and surface charge are also important for the antigen uptake (12, 110).

DCs can uptake antigens by different endocytic pathways: through macropinocytosis where antigens and fluids are taken up forming pinocytic vesicles (4); DCs can also undergo phagocytosis, where particles are engulfed in an actin-dependent process to form a phagosome that interacts with endocytic system to form phagolysosomes that contains an adverse environment against pathogens (121, 135). FcγRs and CLRs play a pivotal role in the antigen uptake and are surface receptors from DCs (136). Phagocytosis may be mediated by receptors such as TLRs and FcγRs, leading to inflammation (137, 138).

Phagocytosis and macropinocytosis are the two main mechanisms by which VLPs are taken up by DCs (136, 139), triggering the innate immune response (2, 34, 113). It has been suggested that the uptake of HIV VLPs by DCs is mediated mainly by macropinocytosis and endocytosis, and it is not mediated by the surface TLR-2 or 4 (28). Similarly, HPV VLPs are taken up by DCs via clathrin and macropinocytosis (actin-dependent process) whereas the uptake of HPV VLPs by LCs is through an actin-independent mechanism (140). The most common pathways of antigen uptake by DCs have been described: phagocytosis, macropinocytosis, and clathrin/caveolae dependent or independent mechanisms (28, 132, 135, 140).

## Processing of VLPs

Upon VLP internalization, activation of PRRs leads to pro-inflammatory responses (131). This response is characterized by the high production of tumor necrosis factor α (TNF-α) and IL-1β, which increases the concentration of proteases in DCs (6). VLPs are processed through the endosomal/lysosomal pathway (2, 6, 15, 23, 25, 42, 48, 95, 112, 141, 142). Lysosomal proteases (such as cathepsins S and D) participate in antigen processing and in the formation of MHCII-peptide complexes (6, 95).

## DC Maturation Induced by VLPs

A very important point is to differentiate between DCs and macrophages. Unlike macrophages, the main function of DCs is not to clear the infection but to prepare the immune system through inflammatory stimuli to recruit inflammatory cells and migrate to lymph nodes to activate T cells (5). DCs are more efficient than macrophages in processing antigens and present them through MHCI (3). Furthermore, lysosomes of macrophages contain very few MHC II molecules and in this place the antigen is digested into amino acids; in contrast, DCs produce high quantities of MHCII molecules which load the antigen-derived peptides (4, 6).

Binding of PAMPs (such as VLPs) to PRRs expressed on the DCs, including TLRs (2, 131) and CLRs (28, 131), prompt the maturation of DCs. During this process, peptide-MHC complexes are produced and lymphocyte costimulatory molecules are expressed on high quantity on the surface of DCs (6, 143, 144) including CD80, CD83, and CD86, which are necessary for efficient activation of effector B and T cells (4, 44, 144, 145). As an example, incubation of immature DCs with HPV-16 VLPs induce overexpression of CD80 and CD83 and secretion of IL-12 (50), which is important for activating Th1 cells (146–148). Similarly, HBcAg VLPs induce the overexpression of costimulatory molecules in DCs such as CD40, CD80, and CD86 (24). Influenza VLPs containing *Giardia lamblia* variant surface proteins increase the expression of CD40 and CD86 signaling through TLR-4 (14).

For DC activation, it is also important the particulate nature of VLPs. Particles are easily taken up by DCs and this event increases the expression of costimulatory molecules in DCs (149). For instance, HCV-VLPs induce overexpression of CD80 and CD86 on DCs after their exposure for 16 h while denatured VLPs (VLPs are generated but denatured by heat treatment) fail to induce this overexpression, indicating that DC activation is mediated by VLPs and not by protein monomers (1). Other examples include: HPV, HIV, Ebola virus, and PPV VLPs which are taken up by DCs inducing their maturation by means of overexpression of costimulatory molecules and production of cytokines (19, 46–48). Similarly, simian HIV (SHIV) VLPs containing HA are taken up by DCs, which overexpress CD40, CD86, and MHC molecules as well as IFN-γ. These DCs induce B and T cell activation as corroborated by target cell lysis and neutralization experiments, respectively (150).

During maturation, DCs also overexpress CCR7 that drives DC migration through lymphatic vessels reaching the T cells in the lymph nodes for their activation (112, 143). However, if the recognition of antigens by T cells is carried out without costimulatory molecules leads to tolerance (103). The maturation process of DCs also includes loss of endocytic activity (151). Finally, maturation process of DCs ends with apoptosis (5).

## Presentation Of VLP Epitopes by DCs

After antigen capture, DCs migrate from non-lymphoid tissues (such as skin and mucosa) to secondary lymphoid organs to present epitopes to T cells (152). There are three types of antigen presentation: through MHCI, MHCII, and cross-presentation. The presentation pathway through MHCI is very important since it can activate CD8^+^ T cells directing them against intracellular pathogens and cancer cells (2, 23, 25, 142). Peptides reaching the cytoplasm will be loaded in the endoplasmic reticulum (ER) and bind MHCI molecules (153).

The other presentation pathway is through MHCII. This presentation pathway depends on the processing of the invariant chain (li) in the endo-lysosomal compartment where peptides of 3 kD are generated (class II associated li derived peptides; CLIPs) (6). CLIPs are bound to MHCII binding groove until their exchange for immunogenic peptides (6), and then, peptide-MHCII complexes are moved to the cell surface of DCs for stimulating CD4^+^ T cells (2, 4, 6, 12, 23, 25, 136, 142, 148, 153).

For cross-presentation, antigens are degraded by the proteasome in the cytosol generating short peptides, and later these peptides are translocated into ER by TAP (transporter associated with antigen presentation) and loaded on phagosomal MHCI molecules (153–155). Later, MHCI-peptide complexes are formed and these are moved to the cell surface (153, 155). The recognition of antigens by CD8^+^ T cells is dependent on the presentation of peptides bound to MHCI molecules on DCs (155).

VLP-derived peptides are presented both trough MHCI and MHCII molecules by DCs (22, 32). For instance, HIV VLPs are taken up and presented by DCs through MHCI and MHCII molecules promoting CD8^+^ and CD4^+^ T cell responses. This is a favorable result, because immune responses based on CD8^+^ T cell are important to eliminate HIV infections (32). Another example includes the HCV VLPs which lead to antigen processing and presentation through MHCI and MHCII molecules. Peptides derived from HCV VLPs are presented to CD4^+^ and CD8^+^ T cells specific for the HCV core protein (1).

PPV VLPs have been designed for expressing exogenous CD8^+^ T cell epitopes (46–48), and it has been shown that these VLPs are taken up by CD8α^+^ and CD8α^−^ DCs isolated from spleen which process these VLPs through proteasome activity and present the T-cell epitopes by cross-presentation using an endosome-to-cytosol pathway (48, 96).

Although it had been suggested that only CD8α^+^ DCs can cross-present antigens, it has been demonstrated that both DC derived from monocytes and LCs are also able to prompt IFN-γ production and cross-present antigens of VLPs to CD8^+^ T cells, although LCs need greater amounts of antigen to do so (107) (Table 1 summarizes the immune response produced during and after the interaction between DCs and VLPs).

**Table 1:** DC PRRs that interact with proteins forming VLPs and prompt an immune response.

**VLP protein bound**	**Target PRR**	**Innate/adaptive immune responses**	**References**
gp120 from HIV	DC-SIGN	Prompt the transmission of HIV from DCs to T cells in wild type HIV. In addition, the interaction of gp120 and DC-SIGN favors a T helper (Th) immune response. SHIV VLPs induce both humoral and cellular immune responses	(42–45, 125, 150)
Protein L1 from HPV	Heparan sulfate, FcγR and DC-SIGN (in LC the PRR is Langerin)	Binding of VLPs to FcγR/DC-SIGN induces high expression of MHCI and costimulatory molecules on DCs as well as cytokine release. As a result, these VLPs induce CTL responses	(7, 15, 44, 107)
Glycoprotein B from CMV	DC-SIGN, TLR2	Glycoprotein B from CMV facilitates the infection of target cells	(44, 106, 123, 124)
E2 protein from HCV	Neither CLRs, TLR2, nor TLR4 are related with VLP binding	VLPs containing E2 protein bind to cell surface of DCs. Antigen processing and presentation through MHCII and cross-presentation lead to CD4^+^ and CD8^+^ T cell responses	(1)
Protein E from dengue virus	DC-SIGN	Protein E from dengue virus binds DC-SIGN from monocyte-derived DCs, reaching lymph nodes, infecting other target cells and later induce high antibody titers	(156–161)
VEE (interaction protein is unknown)	Bind DC and LCs but the receptor of interaction is unknown	VEE target DCs and LCs of skin, migrate to lymph nodes, and reach central nervous system. VEE VLPs induce high neutralizing antibody titers and protect against joint inflammation	(162–164)
Ebola glycoprotein GP (VP40) (similar for Marbug VLPs)	DC-SIGN	Ebola impairs cytokine production and DC maturation but Ebola VLPs prompt the expression of costimulatory molecules, cytokine production and both MHCI and MHCII presentation. Therefore, these VLPs are good candidates to induce humoral and cellular immune responses, but mainly Th1 responses	(165–170)
Protein S from SARS-CoV (and probably SARS-CoV-2)	DC-SIGN	SARS-CoV bind DC-SIGN from DCs without replication in these cells but it favors its transmission to target cells. SARS-CoV VLPs induce costimulatory molecules and cytokines, CD4^+^ T and B cell responses	(38, 171, 172)
Bacteriophage AP205 (AP205 coat protein)	TLR3, 7/8	DCs uptake bacteriophage AP205. After that, TLR3, 7/8 signal pathways are activated and bacteriophage epitopes (and epitopes carried by it) are present in MHCII molecules. This bacteriophage also activates B cells	(40)
Bacteriophage Q-beta (Q-beta protein)	TLR-9	Q-beta VLPs encapsulate CpGs and induce peptide-specific CD8^+^ T cell effective against fibrosarcoma. These VLPs also induces a Th1 immune response as well as class switch from IgE to IgG	(8, 39)

**Figure 2:**
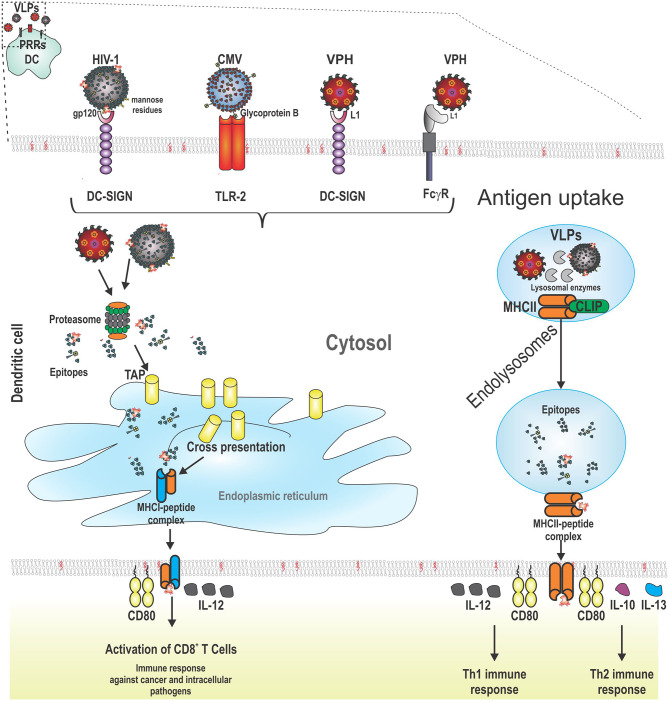
Schematic representation of the interaction between pattern recognition receptors (PRRs) from dendritic cells (DCs) and VLPs. VLPs are recognized by PRRs from DCs (CLRs, TLRs, and FCγR). After this, VLPs are taken up by receptor-mediated endocytosis. VLPs are processed for the presentation of their epitopes through cross-presentation or MHCII. For cross-presentation, VLPs reach the proteasome, are degraded into the cytosol, and the short peptides derived from VLPs are transported to the endoplasmic reticulum by TAP (transporter associated with antigen presentation). MHCI-epitope complexes are moved to the cell surface from DCs to stimulate CD8^+^ T cells which induce lysis of cancer cells or cells infected by intracellular pathogens. The other pathway for antigen presentation is through MHCII molecules. In this case, VLPs are processed into short peptides by lysosomal proteases. In the meantime, the variant chain (li) is processed and loaded into MHCII molecules (class II associated li derived peptides, CLIPs) until their exchange by VLP epitopes. Later, the MCHII-epitope complexes are moved to the cell surface from DCs to stimulate CD4^+^ T cells which help B cells to produce antibodies or to CD8^+^ T cells to enhance their cytotoxicity. Additionally, two signals are required for full activation of adaptive immune system cells: costimulatory molecules (such as CD80) and cytokines (such as IL-12 for CD8^+^ T cells and Th1 cells; and finally, IL-10 and IL-13 for Th2 cells).

In contrast, other DCs such as pDCs bind HPV VLPs and internalize them, but do not undergo maturation (173). Some VLPs do not have the ability to prompt maturation or only induce low levels of MHC molecules. For increasing the immunogenicity of these VLPs, the appropriate selection of an adjuvant is highly recommended. For instance, HCV VLPs are captured efficiently by CD11c^+^ DCs. When these VLPs are co-administrated with dipalmitoyl-S-glyceryl-cysteine (Pam_2_Cys) lipopeptide, DCs increase the expression of MHCII molecules and elicit the production of neutralizing antibodies and CTL responses against HCV. These immune responses were higher than that elicited with HCV VLPs co-administrated with aluminum hydroxide (174) (Figure 2 shows VLPs being taken up by a DC following PRR binding, as well as the processing and presentation of VLP epitopes).

## T Helper (Th) Cell Activation by DCs Loaded With VLP Epitopes

As we mentioned above, VLPs are internalized by DCs by means of different uptake mechanisms and processed for presentation through MHCI and MHCII molecules. T cell activation differs from B cell because T-cell receptors (TCRs) only recognize short peptides bound to MHC molecules. CD4^+^ T cells are called Th cells because they help both B cells and CD8^+^ T cells (2, 24, 175). When B cells need the help of Th cells, this immune response is called T-cell dependent (152), whereas when B cells produce antibodies without T-cells, this immune response is called T-cell independent (2, 12, 46).

Overall, Th cells are essential for generating immune responses based on antibodies and CTL (176, 177). Besides, several works link the Th response to IgG isotypes, because it was demonstrated several decades ago that Th1 and Th2 induces IgG2a and IgG1 immunoglobulin isotypes in mice, respectively (13, 178–180). For instance, immunization of influenza VLPs induces IgG2a and IgG2b anti-HA antibodies strongly suggesting the participation of a Th1 response (11, 14). HCV VLPs also induce Th responses (1).

However, it has been observed that this is not a rule, because some VLPs can induce a strong signal to induce DC maturation as well as cytokine production, which results on T and B cell immune responses without Th cells. This atypical phenomenon has also been observed in the immune response prompted by self-aggregating peptides used as antigen delivery systems (180, 181). It is clear now, that under certain circumstances, Th cells are not necessary for inducing CTL (46, 150, 182) or antibody responses (2, 12, 46, 150). Some VLPs have been shown to provide enough immunogenicity to induce both CTL and antibody responses without Th cells. For instance, immunization of CD4^+^ T cell knockout (KO) mice with chimeric SHIV VLPs coupled to HA from influenza virus induced higher titers of IgA and IgG (including IgG2a) than CD4^+^ T cell KO mice immunized with SHIV VLPs alone. These antibodies had a neutralizing effect. In addition, splenocytes from CD4 KO mice immunized with chimeric SHIV VLPs containing HA resulted in more lysis of cells expressing proteins from HIV than splenocytes of CD4 KO mice immunized with SHIV VLPs alone (150). Thus, the use of HA from influenza contained in SHIV VLPs increases their immunogenicity even in absence of Th cells.

Cytokines play a pivotal role in the Th immune response, mainly IL-12 and IL-10 (146, 148, 183). DCs produce only transiently IL-12 and its availability in the lymph nodes depends on recently activated DCs. Constant levels of IL-12 generate a Th1 immune response (146, 148). However, when levels of IL-12 cease, DCs become exhausted generating a Th2 immune response (146). A strong inhibitor of IL-12 is IL-10 (147, 184). DCs cultivated in the presence of IL-10 can maturate, but cannot induce a Th1 immune response nor induce synthesizes of IFN-γ, favoring a Th2 response (184, 185).

An important DC population that produces IL-12 under microbial stimuli conditions are CD8α^+^ DCs (and sometimes, CD8α^−^ DCs) while pDCs produce both IL-12 and IFN-α in response to several virus infections. IFN-γ enhances the production of IL-12 during inflammatory responses. Interestingly, IL-4 and IL-13 (Th2 cytokines) also induce the production of IL-12 (147). A potent inductor of IL-12 is the lipopolysaccharide (LPS) which activates the nuclear factor kappa B (NF-κB) (184). Nowadays, adjuvants derived from LPS are being used in VLP-based vaccines (119).

## Induction of Cytotoxic T Cell Response by DCs Loaded With VLPs

Unlike Th cells, CD8^+^ T cells recognize peptide antigens charged in MHCI molecules. It was generally accepted that CD8^+^ T-cell responses are prompted only by antigens produced by the DC (endogenous pathway), as occur during infection of intracellular pathogens (48, 186). However, it is now established that some exogenous antigens such as VLPs may access to MHCI pathway and prime CD8^+^ T cells (25, 28, 48, 136).

HCV VLPs are processed and presented by DCs to activate CD8^+^ T cells by cross-presentation (1). Similarly, epitopes associated to RHDV VLPs are processed and charged in MHCI into the endo-lysosome compartments, and recycled to the plasma membrane (136). CD8α^+^ DCs are necessary for cross-presentation of exogenous antigens in mice (12, 48, 96), although other DCs subsets such as pDCs and CD8α^−^ DCs can cross-present too, but less efficiently (187). For instance, PPV VLPs carrying a foreign CD8^+^ T cell epitope (peptide comprising 257–264 of ovalbumin) induced a strong immune response based on CTL without adjuvant use. These VLPs were captured by CD8α^−^ and CD8α^+^ DCs where macropinocytosis and lipid rafts participate. Processing of these VLPs requires vacuolar acidification, proteasome activity, TAP translocation, and synthesis of MHCI molecules (48).

VLPs have been used for inducing CTL responses against exogenous antigens. HBcAg VLPs were modified for containing an epitope of LCMV (p33-VLP). It was observed that DCs of mice injected with these VLPs carried out cross-presentation. For this process, macrophages cross-presented p33-VLP efficiently in the absence of TAP-1, while the cross-presentation was reduced in TAP-1 deficient DCs (188).

For influenza virus, the protective immune response is based mainly in neutralizing antibodies (14). However, although CTL do not prevent influenza infection, the immune response based on CD8^+^ T cells reduces the severity of symptoms and prompt elimination of infected cells (11). VLPs containing influenza proteins induce anti-HA neutralizing antibodies and CD8^+^ T cell responses that produce IFN-γ and lyse infected cells (11, 14).

A very important point to consider is the immunization route. After oral immunization with PPV VLP containing an epitope of LCMV, mice did not generate CTL responses. However, a strong CTL response specific for this epitope was observed in intranasally immunized mice (46, 47). Mice developed a complete protection after challenge with LCMV as well as an effective protection (about 60%) at day 70. Surprisingly, this CTL response was Th cell independent (46).

## Activation of B Cells by VLPs

VLPs have identic conformation to wild type viruses (1, 2). B cells can play the function of DCs, directly recognizing antigens (4). VLPs can prompt the direct cross-linking of B-cell receptors (BCR) (2, 18, 20, 22–24) favoring its capture and transport via the blood stream until reach the spleen, where antigen is delivered to follicular DCs (21). Cross-linking of BCRs also prompts endocytosis of VLPs (24), the B-cell proliferation and increases the production of MHCII and costimulatory molecules (16, 19). Activated B cells interact with Th cells and this allows the immunoglobulin class switch toward IgG and the generation of memory B cells (16). In conditions of strong stimulation of B cells, VLPs can elicit a T-cell independent humoral immune response based on IgM and IgG (2, 31, 150).

VLPs are nanoscale antigen delivery systems that favor the contact of antigens and follicular B cells. Owing to nanoparticles up to 200 nm directly reach the lymph nodes some hours after injection, moving through lymphatic vessels without help of any APC, they can reach B cells in secondary lymphoid organs, persisting in germinal centers on the surface of follicular DCs for several weeks. These nanoparticles induce higher IgG titers than soluble antigens (12, 35, 189). Trapping of nanoparticles is facilitated by complement or natural IgM antibodies (12, 100, 190), facilitating the trapping of particles in lymph nodes (12, 190). Antibody-antigen complexes are antigen depots that maintain the response of B cells for weeks and are appropriate for prompting memory B cells and long-lived plasma cells. If the antigen depot is eliminated, no antibody responses or long-lived memory B cells are generated (12, 191). IgM receptor has been demonstrated in DC, T cells and in B cells mainly (192). DC and activated T cells prompt B-cell differentiation, proliferation, and immunoglobulin production through secretion of cytokines and CD40L (193). Likewise, some VLPs (such as HPV16) induce the production of IFN-α and IL-6 by pDCs. These cytokines play a pivotal role in the production of antibodies against viruses (173). Similarly, as occur in the CTL responses, the immunization route also plays a pivotal role in the antibody production during administration of VLP-based vaccines. Oral immunization with PPV VLPs do not generate antibodies whereas intranasal or parenteral immunization (without adjuvant) generate high antibody levels based on IgG and IgA in the serum and in bronchoalveolar fluid, as well as IgA in feces. These antibodies are able to neutralize PPV infection (47).

## VLPs Under Development Against Tropical and Emerging Viral Diseases

Tropical and emerging viral diseases are a major concern, as they affect the lives of more than a billion people worldwide, mainly low-income populations which are the most vulnerable. Unfortunately the development of vaccines against these tropical diseases has been neglected because they do not represent a relevant profit to pharmaceutical companies. Plant-produced VLPs has been shown to be a promising tool against tropical viral diseases, as they provide thermostability, antigenicity, immunogenicity, and an excellent margin of safety (41).

### VLP-based Vaccines Against Flavivirus

Dengue fever is one of the most important diseases caused by flavivirus. Flaviviruses use their envelope glycoprotein to enter target cells, specifically through its domain III (EDIII), which is also the target of neutralizing antibodies (194, 195). There are four dengue virus serotypes. Primary infection by dengue virus generates a long-lasting and protective immune response against the same serotype, while secondary infection with a different serotype generates a severe disease due to antibody-dependent enhancement through the monocyte and macrophage Fc receptors, leading to the pathogenesis of the disease (156, 196–198). Monocyte-derived DCs are more susceptible to dengue virus infection through DC-SIGN (156–159). In contrast, pDCs do not express DC-SIGN but can recognize dengue virions through activation of TLR7 to produce IFN-α. Virions containing the pre-membrane protein (prM) generate a higher IFN response than virions containing the mature M form. A high IFN-α response and an increase of pDCs correlate with protection. Non-structural proteins from dengue virus alter the signaling cascade involved in IFN-α/β production (156).

An adequate vaccine against dengue virus must generate an effective immune response against all 4 serotypes (197–199). There is a commercial dengue virus vaccine based on a chimeric yellow fever virus designed against all 4 dengue serotypes (197, 200). However, severe side effects have been observed (200). Dengue VLPs have been developed producing their structural proteins (198), but they can also be produced only by expression of envelope protein (197, 199, 201, 202). For the rational design of VLPs against dengue, it is very important to keep in mind that prM proteins are related to disease enhancement (197, 203). Other VLPs formed by HBcAg containing the EDIII antigen have been produced as a fusion protein, but unfortunately these VLPs induced low neutralizing antibody titers (194). The most efficient VLP-based vaccine against dengue fever was designed containing the DSV4 antigen (the EDIII of the envelope protein of the 4 dengue serotypes), which was fused to the HBsAg and co-expressed with the free S antigen to form VLPs. DSV4 VLPs induced neutralizing antibodies against the 4 dengue serotypes in mice and macaques, eliminating viremia and protecting after challenge. Furthermore, in a mouse model of disease enhance, mice and macaques antibodies against DSV4 did not induce inflammation or mortality (204). Similarly, other dengue VLPs were produced co-assembling the envelope protein of the 4 dengue serotypes. Mice immunized with 3 doses of these VLPs developed neutralizing antibodies against all 4 dengue serotypes (203).

Zika virus is another important flavivirus, its infection is generally asymptomatic or produces mild disease, but during pregnancy, it is associated with defects at birth and in infants it produces visual and hearing defects (205–207). Zika VLPs have been developed by co-expressing structural and non-structural proteins including CprME and NS2B/NS3, respectively. These VLPs promote higher antibody titers than those promoted with an inactivated vaccine (205). Zika VLPs have been shown to confer protection in mouse models in which zika is fatal (AG129 strain, IFN-α, β, and γ receptor null) and reduce viremia in other mouse models (BALB/c strain) presumably due to neutralizing antibodies, as indicated by a passive transfer neutralization assay (207), although memory T cells could also play an important role (206).

Japanese encephalitis is also important, and as it occur with other flavivirus, the envelope glycoprotein is important for the attachment and entry of the virus into target cells (208). Expression of the envelope glycoprotein generates VLPs, and co-expression of prM is not necessary (208–210). Although there is an authorized vaccine (JE-VAX) based on an inactivated virus produced in brain cells, its main drawback is the development of allergy to brain proteins or even prion contamination, besides its high cost (208). A singe immunization with these Japanese encephalitis VLPs without adjuvant co-administration can protect 50% of challenged mice whereas with adjuvant [poly(γ-glutamic acid) nanoparticles or aluminum] it can protect >90% (211), but can reach up to 100% protection (in mice immunized intraperitoneally twice) (208). Mice protection correlates with high neutralizing antibody titers that last for at least 6 months (211). Japanese encephalitis VLPs have also been produced as a chimeric VLP inserting foreign epitopes in several loops without damaging the self-assembly process (212).

In the case of West Nile virus (WNV), its infection is facilitated by endothelial cells until it reaches the nervous system (213). WNV infects birds and mammals (214), there are WNV authorized vaccines for horses (213, 215, 216), whereas human vaccines are in clinical trials (217). WNV infects LCs prompting its transport to lymph nodes (218); but WNV can also be detected in spleen (219, 220). The αVβ3 integrin is the main potential receptor for WNV. WNV protein E, particularly EDIII, mediates cell receptor attachment and endocytosis (214).

### VLP-based Vaccines Against Togavirus

Chikungunya and Venezuelan equine encephalitis (VEE) viruses are also transmitted by mosquito bites (162, 221). Chikungunya virus cannot bind or replicate in DCs or lymphocytes, but it replicates in endothelial cells, fibroblasts and monocyte-derived macrophages (160, 221). Live attenuated vaccines against chikungunya have been shown to be immunogenic in clinical trials but they also produce side effects like arthralgia (222). Unlike, chikungunya VLPs generated by the expression of their structural polyprotein (C, E3, E2, 6K, E1) demonstrated to elicit neutralizing antibodies, reduce the viremia and protection against joint inflammation after mice (222) and non-human primates challenge (163).

VEE virus targets DCs and LCs resident of skin; upon infection, these APCs migrate to lymph nodes and later infect the central nervous system leading to lethal encephalitis in horses. In humans, VEE virus produces flu-like symptoms, although encephalitis is also observed in older people and children (162, 164). VEE virus also infects efficiently osteoblasts (164). There are not effective vaccines against VEE virus, but monovalent and polyvalent equine encephalitis VLPs (including western and eastern equine encephalitis) have been produced. These VLP-based vaccines induce protective neutralizing antibodies as indicated by an IgG passive transfer experiment against an aerosol challenge (223).

### VLP-based Vaccines Against Bunyavirus

VLPs against emerging diseases such as Crimean-Congo Hemorrhagic Fever (CCHF) and hantavirus have also been produced. CCHF causes hemorrhagic fever in humans, with high mortality. CCHF VLPs were developed expressing the glycoproteins Gc and Gn, the viral polymerase and the nucleoprotein encapsulating a minigenome. Immunization with these VLPs has been shown to induce 100% protection in challenge with transgenic mice [A129 strain, STAT1 (signal transducer and activator of transcription 1) null]. The immune response generated was humoral and cellular, but dominant Th1 and balanced Th2 responses correlated with protection (224). These VLPs have also been used in ELISA (enzyme linked immunosorbent assay) and neutralization assays (225). Similarly, hantavirus VLPs have also been designed co-expressing the nucleocapsid protein, and glycoproteins Gn and Gc in Chinese hamster ovary cells. These VLPs produce both humoral and cellular immune responses against hantavirus (226). Rift Valley fever virus (RVFV) in another important bunyavirus which affects humans and livestock generating fever, meningoencephalitis, hepatitis, and severe hemorrhagic disease (227). RVF VLPs are produced by expressing the polymerase L, the nucleocapsid protein N and the M protein (227, 228). As with other VLPs derived from bunyaviruses, these VLPs demonstrated to induce protective neutralizing antibodies (228).

### VLP-based Vaccines Against Filovirus

Ebola and Marbug viruses are filoviruses that cause hemorrhagic fever reaching high mortality rates (165, 166). These filoviruses replicate in DCs, do not induce cytokine production or DC maturation (166, 167). Ebola VLPs can be produced expressing their glycoprotein GP and matrix protein (VP40) (165, 168). Immunogenicity evaluated *in vitro* by using DCs indicated that these VLPs induces the expression of CD80, CD86, MHCI, MHCII as well as cytokines IL-6 and IL-10, primordially (165, 169). Ebola and Marbug VLPs also induce TNF-α and IL-8 and stimulate T cell responses (166). Mice immunized with Ebola VLPs generate neutralizing antibodies that correlate with 100% protection (165, 169). Antibodies generated with Ebola VLPs are IgG2a subtype strongly suggesting a Th1 immune response (168).

### VLP-based Vaccines Against Coronavirus

Nowadays, coronaviruses have become pathogens of great importance due to their ability to cause pandemics. Severe acute respiratory syndrome (SARS) is caused by the coronavirus SARS-CoV. SARS-CoV binds to angiotensin-converting enzyme 2 (ACE-2) through their receptor-binding domain (RBD) found in the S1 region of the spike (S) protein (38). SARS-CoV targets DCs through the interaction of S protein with DC-SIGN, but although it cannot replicate in DCs, it can promote its transmission to other target cells (171).

SARS-CoV VLPs can be produced expressing membrane, envelope, and S proteins through co-infection of recombinant baculoviruses (38, 229). In DCs, these VLPs increase the expression of costimulatory molecules: CD80, CD83, and CD86 as well as the secretion of IL-6, IL-10, and TNF-α. Additionally, co-culture of CD4^+^ T cells and DCs stimulated with SARS-CoV VLPs induces IFN-γ and IL-4 synthesis by CD4^+^ T cells (38). For mucosal immunity, the route of administration of vaccines plays a pivotal role: mice immunized with SARS-CoV VLPs via the intraperitoneal route produced both cellular and humoral immune responses, with the humoral immune response based on neutralizing IgG. VLPs administrated via intranasal route produced lower levels of IgG but higher levels of sIgA (172). More recently, a novel coronavirus emerged in China. This coronavirus is now called SARS-CoV-2, and uses the same cell receptor ACE2 as that for SARS-CoV (230). SARS-CoV-2 produces the disease known as Covid-19, which is a current pandemic responsible for the infection of millions of people worldwide and several hundred thousand deaths to date. A new vaccine based on VLPs could be developed to help counter this pandemic.

## VLPs as Immunotherapy Platforms Against Allergic Diseases

VLPs are important tools in preventive medicine but they have also been used in treatments against allergic rhinitis and asthma (8, 9, 23, 58, 231, 232). VLPs are good candidates for allergy therapy due to several reasons. VLPs can be administrated together with TLR ligands forming structures readily recognized by TLRs from DCs (8, 23). VLP-based vaccines against allergic diseases allow the allergen antigen presentation and prompt Th1 immune responses (8, 23). It is very important to note that anti-allergic vaccines direct the immune response to a Th1 profile, since molecules related to a Th2 profile such as IL-4, IL-5, IL-9, and IL-13 as well as IgE are involved in asthmatic problems (58, 232).

The VLPs most frequently used in this field are based on bacteriophage Q-beta (8, 9, 23). The design of these VLPs is based on the chemical attachment of epitopes from allergens on the surface of Q-beta VLPs. Subcutaneous or intramuscular immunizations of these VLPs induce high IgG titers against allergens without adjuvant co-administration and without inducing IgE (18). However, some adjuvants can also be used for the improvement of the anti-allergic effect of vaccines based on VLPs. VLPs and adjuvants such as CpG motifs, can be used in anti-allergic vaccines even without the allergen (9, 23).

The anti-allergic mechanism of Q-beta VLPs containing CpG motifs is explained at the DC level. TLR-9 of pDCs is activated by CpG motifs encapsulated by Q-beta VLPs (9). This leads to the production and secretion of Th1 cytokines which promote the IgG class switch (23, 233). That is why, CpG motifs are adjuvants frequently used in anti-allergic therapies. There are several types of CpG motifs: type A CpG induces IFN-α in pDCs whereas type B CpG induces IL-12 in conventional DCs which inhibits the development of Th2 responses and prompt Th1 responses, inhibiting IgE synthesis and reducing the allergic activity of mast cells, basophils and eosinophils (8, 9, 23, 58, 231, 232). CpG motifs have been encapsulated into VLPs for two reasons: to protect the DNA of degradation and improve the efficient delivery on pDCs. Asthmatic patients immunized with VLPs based on bacteriophage Q-beta encapsulating type A CpG have less asthmatic symptoms than patients injected with placebo (58). Besides, type B CpG induces the expression of inducible costimulator ligand (ICOS-L) on pDCs which is important for promoting regulatory T cells (23, 234). T cells primed by pDCs stimulated by type B CpG produce high quantities of IL-10, and a moderate quantity of IFN-γ and TNF-α (231, 234).

VLPs have been shown to be good anti-allergic vaccine candidates for treating patients allergic to the house dust mite (9, 23). For instance, a peptide comprising 16 amino acids of the house dust mite was chemically coupled to Q-beta VLPs loaded with RNA (Q-beta self-assemble in presence of nucleic acids) and it was observed a rapid humoral immune response based on IgM and IgG against both the 16 amino acid peptide and VLPs, the only side effect observed was a mild skin reaction. In addition, no increase in IgE titers was observed (18, 23).

Due to the fact that immunostimulatory molecules such as CpG motifs alleviate atopic and allergic diseases in patients, encapsulation of CpGs into VLPs containing allergens is a good strategy for targeting pDCs to improve the health of these patients (58). Besides, allergens coupled to VLPs don not promote anaphylactic reactions (23, 235), instead they act as adjuvants facilitating antigen presentation and enhancing the Th1 immune response (9, 23). In addition, bacteriophage Q-beta nanoparticles trapping nucleic acids that work as TLR-9 ligands are efficiently taken up by pDCs and prompt the antibody production or regulatory T cells depending on the type of CpG motif used. This represents a safe platform for the generation of anti-allergic vaccines.

## VLPs as Platforms for Cancer Vaccines

Many viruses are involved in cancer, some examples are: HPV, HBV, and HCV, which are oncoviruses leading to diseases such as cervical cancer and hepatocellular carcinoma, respectively. VLPs based on these viruses possess anti-cancer properties per se (34, 236). HPV VLPs containing L1/L2-E7 proteins of HPV induce CTL responses against a tumor cell line expressing E7 protein (19, 237). This is an evidence of the generation of adequate costimulatory signals provided by mature DCs. Similar responses have been observed on PPV VLPs (19, 46).

Due to the high immunogenicity of VLPs, they can be used as scaffolds for designing vaccines against other types of cancer. One of the main strategies to do that is the chemical conjugation of cancer epitopes on the surface of VLPs, although genetic fusion has also provided excellent results (51, 52, 236). VLPs containing cancer epitopes are taken up by DCs, processed and presented to Th cells as well as cytotoxic T cells (236), as with other epitopes exposed on VLPs (2, 23, 25, 142). Some examples include: murine polyomavirus displaying the prostate specific antigens (proteins overexpressed in prostate cancer cells) (236, 238). The same VLP platform was used to develop a vaccine against breast cancer (fusion of VLPs with the antigen Her2). In this case, strong CTL responses against antigen Her2 were observed, although weak humoral responses were also observed (236, 237, 239). Similarly, influenza VLPs containing Her2 have been used as vaccine against breast cancer, but in this case, both cellular and humoral immune responses have been observed (236, 240). Other examples include bacteriophage Q-beta and HBV VLPs, which have been used to design a vaccine against melanoma (containing the melanoma associated antigen 3) (23, 57, 236).

Cancer vaccines need to induce Th1 immunity in order to activate CTL responses which target and lysate cancer cells (142, 236). TLR ligands are used to activate DCs, promote their maturation, cytokine production and activation of CTLs (56, 57). In a very interesting work, a VLP-based vaccine against melanoma was designed. CpG motifs were encapsulated into Q-beta VLPs containing the Melan-A/Mart-1 peptide which is specific to melanoma cells (56, 57). Melanoma patients were immunized with these VLPs and it was showed that they generated CTLs able to degranulate and produce Th1 cytokines (56, 57). Similarly, RHDV VLPs and the coupled heterologous antigens can be presented by DCs for inducing CTL responses (83). Besides, anti-cancer drugs such as doxorubicin can also be encapsulated into modified VLPs (51) (the reader is referred to an extended review about VLPs used as cancer vaccine platform published by Ong et al. (236).

## Advantages of VLPs

Attenuated pathogens found in traditional vaccines have helped to control and even eradicate several infectious diseases. However, pathogens contained in these vaccines can replicate into the host cells and can spread in the population or recombine resulting in new pathogenic strains (180, 241). Like traditional vaccines, VLPs are immunogenic and excellent to control infectious diseases. One of the main advantages of VLPs is safety. VLPs cannot replicate, recombine or undergo reassortment because they do not contain infectious DNA or RNA material; therefore, VLPs are safer than traditional vaccines (2, 180). For instance, in the case of veterinary vaccines, a commercial vaccine based on a chimeric PCV1/2 was found to infect and spread in vaccinated pigs in Canada (75, 242). This vaccine had to be taken off the market for safety reasons (75). Situations like this are not a concern when VLPs-based vaccines are used instead.

VLP-based commercially available vaccines are produced in yeast (25, 34) and baculovirus expression systems (25). Baculoviruses are often found in vegetables and fortunately, they are not capable to replicate in mammalian cells (31); therefore, they are not pathogenic for mammals (243).

Several VLPs are immunogenic *per se*, and adjuvants are not needed. However, TLR ligands (9, 19, 23, 24, 58, 231, 234) as well as recombinant immune complexes can be used to increase their immunogenicity; the last one has been shown to elicit a very strong antibody response (titers >1:1,000,000) (62). Modifications of VLPs can also be done to increase their stability and adjuvant effect. For instance, retrovirus VLPs decorated with the variant surface proteins from *Giardia lamblia* (which are resistant to proteolytic digestion and are ligands of TLR-4) have been designed. Mice immunized with these VLPs containing HA were protected from the challenge. Additionally, western blot analysis showed that these VLPs withstand to proteolytic digestion, adverse pH and temperatures (14).

Other VLPs are based on influenza virus. Subunit influenza vaccines have a good genetic match to the original isolated virus, are frequently produced in the baculovirus expression system, so that they do not contain egg-proteins (244), they have high yields and are inexpensive (11). Other way to produce influenza vaccines include those produced in eggs. Unfortunately, influenza virus produced in eggs has genetic changes due to adaptation to chicken embryos as well as it contains egg proteins (245, 246). Egg proteins may induce allergies, which limit the use of egg-produced vaccines (11).

Besides, influenza proteins can be used to improve the binding efficiency of other VLPs. For example, chimeric HA/SHIV VLPs can bind to DCs stronger than SHIV VLPs and this has been correlated with stronger antibody and T-cell responses (150).

## Limitations Of VLPs

Currently, VLPs are as good as traditional vaccines but with the advantage of being safer. However, some drawbacks need to be highlighted. Some vaccines based on VLPs are very complex, and therefore, their price is high (62). VLPs require a purification process where the use of density gradients or even chromatography are necessary (29), and therefore, the cost of these downstream processes is high and time consuming (247).

The use of VLPs as antigen carriers can be complicated due to several difficulties: an adequate viral construct must be selected for incorporating proteins or peptides on their structure, a new particle needs to be designed for each disease, the adjuvant effect as well as the inflammatory response must be evaluated before use in each new VLP (142). For the case of chimeric VLPs, the insertion of exogenous peptides is very restrictive. The insertion of more than 20 amino acids may damage the self-assembly properties of VLP proteins. In addition, although the design of chimeric VLPs can be based on the predictions of the structure, this process is often empirical (2).

Several VLPs are produced in the baculovirus expression system, but although baculoviruses do not infect mammals they represent an environmental concern. Infectious baculoviruses may remain in the preparation containing VLPs, that is why baculoviruses are inactivated but this process can damage the immunogenicity of VLPs (2).

VLPs produced in yeast are also safe; however, there has been reports mentioning anaphylactic reactions after immunization with VLP-based vaccines produced in yeast (such as HBV VLPs). This side effect is a rare even in subjects with IgE anti-yeast antibodies (248, 249). In clinical trials, it was reported that about 2% of analyzed subjects had anti-yeast IgE before immunization but these subjects did not increase their IgE levels after immunization (248).

## Discussion and Perspectives

Although VLPs can be internalized by other mechanisms such as macropinocytosis (by its mesoscopic scale) and phagocytosis (e.g., when using a particulate adjuvant), in this work we focus on analyzing the main proteins used to produce VLPs targeting DC through their PRRs, favoring the receptor-dependent endocytosis mechanism.

Here, we analyze several VLPs utilized either as a preventive agent or as a therapeutic agent according to their abilities to prime innate immune responses. The first point to consider for developing smart VLP-based vaccines is based on the selection of a VLP candidate according to the type of immune response that the individual requires. Thus, if it is necessary to prevent any infection by a pathogen that can be blocked by antibodies, the most appropriate VLP should be selected for this case. If the pathogen is intracellular, VLPs targeting DCs that can perform cross-presentation with high efficiency should be selected. For example, RHDV VLPs have been shown to be very effective in carrying cancer epitopes. These VLPs increase the production of MHC molecules as well as costimulatory molecules (83). Therefore, to develop cancer vaccines, these VLPs are an excellent candidate.

Another important point is to evaluate which method is better for the incorporation of exogenous antigens in VLPs. There are two main ways to do it: chemical conjugation and protein fusion. In one work, the cellular immune response generated by two types of RHDV VLPs containing the same antigen was evaluated. In one type of RHDV VLP, the gp33 peptide (derived from choriomeningitis virus) was recombinantly produced as a fusion protein, whereas in the other one, the gp33 peptide was chemically conjugated to the RHDV VLP. RHDV VLPs with gp33 incorporated as a fusion protein generated stronger cytotoxicity than RHDV VLPs chemically conjugated with gp33. Interestingly, these VLPs were internalized by two DC11c^+^ DC subsets: CD8α^+^ and CD8α^−^ (55). Thus, it was suggested that fusion of epitopes to VLP proteins is more efficient than their chemical conjugation. Another important point to consider is the next one: the insertion of epitopes must be carried out in the loop structures of the VLP protein that are not important for oligomerization and the incorporated epitopes must be short.

VLPs can also be modified to increase their uptake and improve the processing of incorporated epitopes. As an example, RHDV VLPs containing a melanoma epitope (KVPRNQDWL derived from glycoprotein 100) were developed. The melanoma epitope containing proteasome cleavable linkers was fused to N-terminus and C-terminus from VP60 protein. These VLPs were also conjugated with mono and dimannosides to favor their internalization. It was observed that these VLPs entered in APCs through more than one pathway, increasing the internalization of the antigen through phagocytosis and macropinocytosis. Furthermore, the use of linkers allowed proper processing of the epitope, as indicated by experiments in mice vaccinated with these VLPs that were challenged with tumor cells after vaccination, where specific lysis, decreased tumor size, as well as increased survival percentage were observed (250). Therefore, VLPs can be modified not only for targeting DCs to program T-cell activation but VLPs can also be modified to be better processed in a more suitable way by the proteasome by using linkers to favor a more precise antigen presentation.

The correct selection of an adjuvant is also important to promote DC maturation for a suitable presentation of the antigen, and therefore, for the generation of a strong adaptive immune response. Although many VLPs do not require adjuvant co-administration, its use can favor the type of response desired. In the case of allergic diseases, in which the response is favored toward a Th2 profile, an adjuvant used for the formulation of VLP-based vaccines must favor a Th1 response as well as the immunoglobulin class switch from IgE to IgG. VLP-based vaccines against allergic diseases are primarily based on bacteriophage Q-beta proteins that assemble in the presence of nucleic acids to form VLPs (8). This property has allowed to encapsulate ligands of TLRs and thus activate DCs for their maturation and antigen presentation to CD4^+^ T cells, production of Th1 cytokines and class switch from IgE to IgG. Similarly, VLP-based vaccines designed against cancer should contribute to restoring the immune response. VLPs co-administrated with a suitable adjuvant activate DCs, promote their maturation and proper presentation of cancer antigens to CD4^+^ and CD8^+^ T cells.

Nowadays, vaccine production against emerging tropical viral diseases is a concern. There are several vaccines produced in a traditional way, including inactivated or attenuated viruses, but many of them have not been commercialized due to safety issues (205, 251), while for other tropical viral diseases there is no vaccine available to this date (206). The smart design of VLPs is not only based on expressing structural proteins to produce VLPs. Mainly, for the design of VLPs against tropical viral diseases, at least three things must be considered: the self-assembling protein, the receptor binding protein (target of neutralizing antibodies, preferably a domain), and avoid the production of proteins involved in pathogenesis of the disease. Some VLPs against tropical viral diseases can be developed in a simple way, expressing only envelope proteins avoiding pre-membrane or membrane proteins. A bad design of VLP sometimes can generate deleterious effects in vaccinated individuals as in the case of dengue virus.

All this knowledge together may be useful to design smart VLPs as a tool for personalized medicine, in which a VLP can be selected in a particular way and modified according to the individual needs of each patient.

The smart design of VLPs could include the insertion of modules in VLP proteins. The modular VLPs design may allow that a single VLP backbone can be decorated with additional proteins as needed for each specific case to incorporate several modules such as: carriers, T and/or B cell epitopes, glycosylated epitopes, epitopes containing proteasome cleavable linkers for improving their processing, and one or several PRR ligands to enhance their uptake by DCs.

Although the main aim of this work was focus on VLP-based vaccines targeting DC PRRs to prompt a strong immunogenicity, modular VLPs could also be designed with DNA binding modules for efficiently carry nucleic acids for transduction of other specific target cells. VLPs containing genes have been designed for *in vitro* transduction of cells (252), but we speculate that they may soon be used in animal models for gene therapy.

## Concluding Remarks

The right design of VLPs should not be based only in the overexpression of structural proteins of viruses. Instead, the smart design of VLPs must be based on the molecular mechanism of infection of the pathogen, the target PRRs to which VLPs will attach for their internalization, and the type of immune response desired. VLPs can also be designed for their use in the treatment of allergic diseases, because the immunization with VLPs can switch the immune response from Th2 to Th1. Since VLPs can restore the immune response, be cross-presented and induce a CTL response, they can also be used for future cancer therapies. The ability to engineer VLPs with exquisite detail makes them a very attractive candidate for the design of platforms aimed at the production of vaccines against infectious pathogens as well as against allergic and cancer diseases used in personalized and/or translational medicine.

## Author Contributions

JZ-C conceived the main idea of this work, conducted the search of bibliography, designed and wrote the most topics of this review. JZ-C, JR-J, and LV wrote the final draft and designed the figures of this review article. LV corrected the manuscript and provided important contributions during the development of this work. All authors approved the final manuscript.

## Conflict of Interest

The authors declare that the research was conducted in the absence of any commercial or financial relationships that could be construed as a potential conflict of interest.

## Abbreviations

ACE-2: angiotensin-converting enzyme 2
APC: antigen presenting cells
AS: adjuvant system
BCR: B-cell receptors
CAV: chicken anemia virus
CCHF: Crimean-Congo Hemorrhagic Fever
CCr7: The CC chemokine receptor 7
CDPs: monocytoid lineage and common DC progenitors
CLIPs: class II associated li derived peptides
CLRs: C-type lectin receptors
CMV: cytomegalovirus
COBRA: computationally optimized broadly reactive antigen
CRD: carbohydrate recognition domains
CTL: cytotoxic T-lymphocyte
DC: dendritic cell
DC-SIGN: DC–specific intercellular adhesion molecule 3–grabbing non-integrin
DSV4 antigen: the EDIII of the envelope protein of the 4 dengue serotypes
EDIII: envelope domain III
ELISA: enzyme linked immunosorbent assay
F: fusion protein
Fl3: FMS-like tyrosine kinase 3 ligand
GM-CSF: granulocyte/macrophage colony-stimulating factor
HA: hemagglutinin
HBcAg: HBV core antigen
HBsAg: HBV surface antigen
HBV: hepatitis B virus
HCV: hepatitis C virus
HIV: human immunodeficiency virus
HN: hemaglutinin-neuraminidase protein
HPV: human papillomavirus
ICOS-L: inducible costimulator-ligand
IFN: interferon
Ig: immunoglobulin
IL: interleukin
IRF: interferon regulator factor
KO: Knockout
LCMV: lymphocytic choriomeningitis virus
LCs: Langerhans cells
li: invariant chain
LPS: lipopolysaccharide
M: membrane protein
MDP: macrophage—dendritic cell progenitor
MHC: major histocompatibility complex
MPLA: monophosphoril lipid A
NA: neuraminidase
NALP3, NACHT, LRR: and PYD domains-containing protein 3
NDV: Newcastle disease virus
NF-κB: nuclear factor kappa B
NLR: NOD-like receptors
NP: nucleocapsid protein
Pam_2_Cys: dipalmitoyl-S-glyceryl-cysteine
PAMPs: Pathogen associated molecular patterns
PCV: porcine circovirus
pDCs: plasmacytoid DCs
PPV: porcine parvovirus
pre-mDCs: predendritic cells
prM: pre-membrane protein
PRR: pattern recognition receptor
RBD: receptor-binding domain
RHDV: rabbit hemorrhagic disease virus
RSV: respiratory syncytial virus
RVFV: Rift Valley fever virus
S antigen: S antigen from HBV
S protein: spike protein
SARS: severe acute respiratory syndrome
SHIV: simian HIV
STAT1: signal transducer and activator of transcription 1
TAP: transporter associated with antigen processing
TCRs: T-cell receptors
TGF-β1: transforming growth factor beta 1
Th cells: T helper cells (CD4^+^)
TLRs: Toll-like receptors
TNF-α: tumor necrosis factor α
VEE: Venezuelan equine encephalitis
VLPs: virus-like particles
WNV: West Nile virus
Zbtb46: zinc finger and BTB domain containing 46.
